# The prevalence of metabolic dysfunction-associated steatotic liver disease (MASLD)-related advanced fibrosis and cirrhosis in the United States population utilizing AGILE 3 + and AGILE 4 scores: analysis of the NHANES 2017–2018 cycle

**DOI:** 10.1186/s12876-024-03295-8

**Published:** 2024-07-08

**Authors:** Naim Alkhouri, Ashraf Almomani, Phuc Le, Julia Y. Payne, Imad Asaad, Prido Polanco, Phillip Leff, Prabhat Kumar, Mazen Noureddin

**Affiliations:** 1 Hepatology and Liver Transplantation, Arizona Liver Health, Chandler, 20201 W Fairview St, AZ 85224 USA; 2 Cleveland Clinic Florida, Florida, USA; 3 Cedar Sinai Medical Center, Los Angeles, CA USA; 4 Cleveland Clinic Foundation, Ohio, USA

**Keywords:** MASLD, AGILE, Elastography, Cirrhosis, Advanced fibrosis, NHANES

## Abstract

**Background:**

Studies attempted to estimate MASLD-related advanced fibrosis (AF) and cirrhosis (MC) prevalence utilized tests with low positive predictive value (PPV) which overestimates prevalence. AGILE3 + and 4 scores were developed to increase the PPV of both; respectively. In this study, we used these scores to assess the prevalence of AF and MC.

**Methods:**

Participants aged ≥ 18 years with VCTE exam in the NHANES 2017–2018 cycle were included. We excluded pregnant women, patients with excessive alcohol intake, hepatitis B/C, and ALT or AST > 500 IU/L. MASLD was defined with CAP score > 248 dB/m. MASLD subjects with AGILE 3 + score of ≥ 0.68 and AGILE 4 score of ≥ 0.57 were considered to have advanced fibrosis and cirrhosis; respectively. AGILE 3 + of 0.45–0.67 and AGILE 4 of 0.25–0.57 were grey zone, whereas AGILE 3 + < 0.45 and AGILE 4 < 0.25 were considered a rule-out.

**Results:**

1244 subjects were included in the final analysis. The Median age was 53 (51.4–54.6) years, 55.6% were male, median BMI was 33.8 kg/m2 and 41.1% had T2DM. Based on AGILE 3+, 80.3% of the MASLD population were at low risk for AF and 11.5% were in grey zone. The prevalence of AF due to MASLD was 8.1% corresponding to 4.5 million Americans. Based on AGILE 4 score, 96.5% of the MASLD population were at low risk for cirrhosis and 2.4% were in the grey zone. The prevalence of MASLD-cirrhosis was 1.1% corresponding to 610,000 Americans.

**Conclusion:**

Our results suggest that approximately 4.5 million people in the U.S. have AF and 0.6 million have cirrhosis due to MASLD.

## Introduction

Metabolic Dysfunction-Associated Steatotic Liver Disease (MASLD), previously known as Non-alcoholic Fatty Liver Disease (NAFLD), is a histological disease spectrum that ranges from fatty liver infiltration to steatohepatitis (previously known as Non-alcoholic Steatohepatitis, MASLD), advanced fibrosis and – eventually- cirrhosis. MASLD is a leading cause of end-stage liver disease worldwide and is predicted to emerge as the sole leading cause in the next few decades, affecting both adults and children [1, 2]. Owing to the obesity pandemic, the global prevalence of MASLD has been on the rise and is currently estimated to be ~ 25% (compared to ~ 15% on 2005). In a recent cross-sectional analysis in the United States, the biopsy-proven prevalence of MASLD in the U.S. adults has been ~ 21.9%, representing 51.6 million potentially affected individuals [3]. Liver biopsy has been the gold standard to determine the prevalence of MASLD fibrosis and cirrhosis; however, several noninvasive tests and scoring systems have been developed to estimate the prevalence of the two entities [5]. These scores include AST to platelet ratio index (APRI) which was originally developed for use among HCV patients and was later validated to predict MASLD-related advanced fibrosis with a sensitivity and specificity of 75% and 86%, respectively [6, 7]. Other scoring systems like the fibrosis-4 index (FIB-4) and the NAFLD fibrosis score (NFS) were also validated in patients with good accuracy [7, 8]. However, owing to relying on routine blood tests alone (Aspartate aminotransferase {AST}, alanine aminotransferase {ALT}, platelets, etc.), the accuracy of these tests can be easily affected by several factors that are not related to liver disease. More importantly, the positive predictive value (PPV) of these tests is relatively low, leading to potentially overestimating the true disease prevalence. For these reasons, AGILE 3 + and AGILE 4 scores have been recently developed by combining routine clinical variables, lab chemistry values and vibration-controlled elastography (VCTE) parameters to specifically increase the PPV of predicting MASLD-related advanced fibrosis and cirrhosis; respectively. Our aim in this study is to estimate the prevalence of MASLD-related advanced fibrosis and cirrhosis using these new scores.

## Methodology

### Database

Established by Center for Disease Control and Prevention (CDC), The National Health and Nutrition Examination Survey (NHANES) database serves as a major national database designed to understand the health and nutritional needs of children and adults in the United States since 1960s. NHANES databases program annually examines a nationally representative sample of ~ 5,000 individuals located in counties across the United States. The survey interview includes demographic, socioeconomic, dietary, and health-related questions. The examination further includes medical, physiological and laboratory measurements performed by highly trained medical personnel at a central laboratory, in addition to interview questionnaires and standardized physical examination. Data from the survey is used in epidemiological and health-related studies which help to design further health programs and services. The survey was approved by the Institutional Review Board at the Center for Disease Control and Prevention, and informed consent was obtained from all participants. Data from NHANES 2017–2018 is the most recent survey cycle that provided transient elastography information as determined by FibroScan® and was utilized for this analysis.

### Definitions and inclusion criteria

**Fig. 1:**
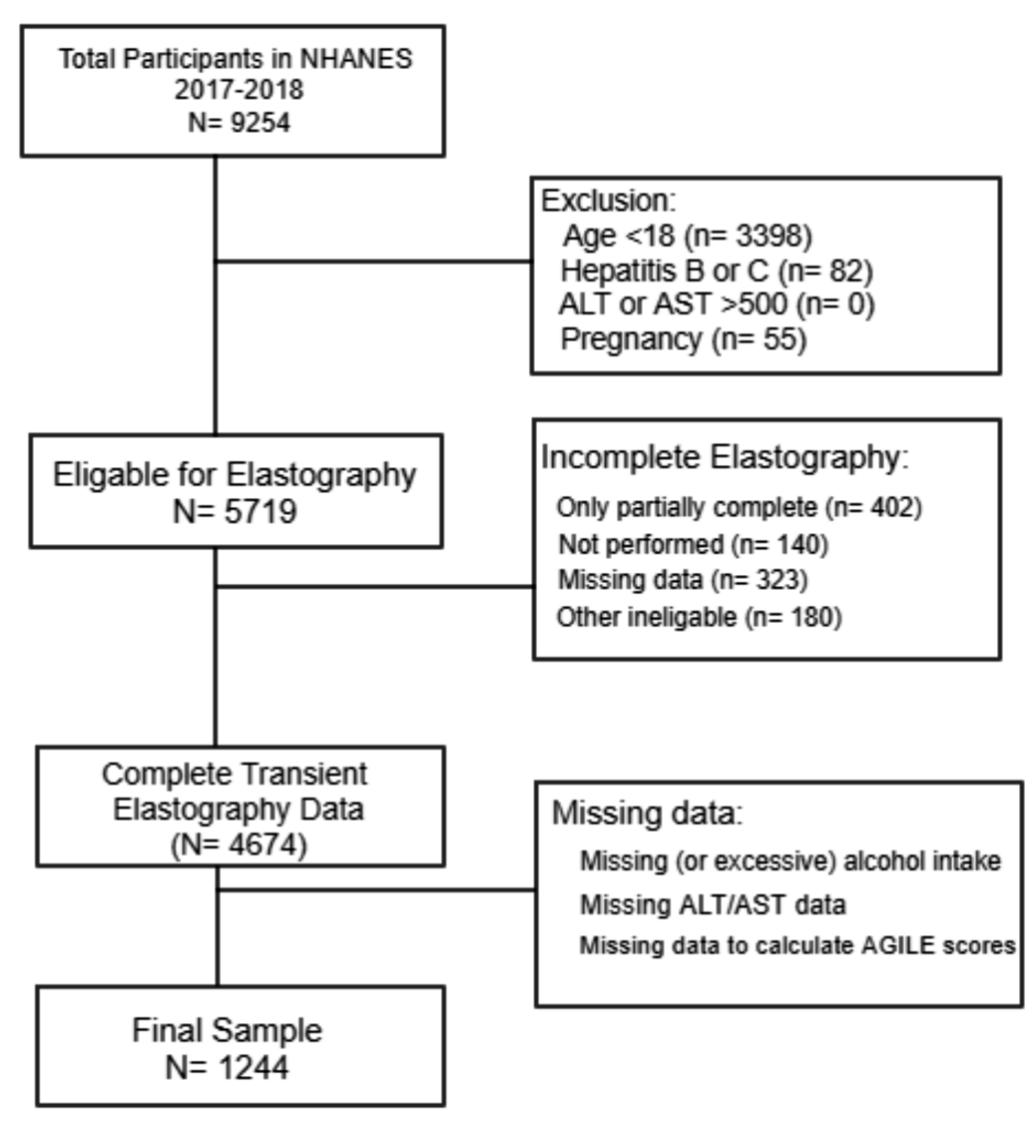
Inclusion criteria and patient selection

NHANES 2017–2018 is the most recent survey cycle that provided transient elastography data. Our study population included participants aged ≥ 18 years old who had a complete transient elastography exam. NHANES used FibroScan® model 502 V2 Touch equipped with medium and extra-large probes. We excluded pregnant women, missing alcohol data, hepatitis B or C, and ALT or AST > 500 IU/L. Among these patients, we stratified based on excessive alcohol consumption defined as > 2 drinks/day for males and > 1 drink/day for females and obesity defined as a Body Mass Index (BMI) of 30 kg/m2 or more. The inclusion criteria and participants stratification algorithm are shown in Fig. 1. We further stratified based on steatosis (CAP ≥ 248 dB/m) to identify MASLD. Our final sample was 1244 participants.

### Statistical analysis

#### Characteristics of study participants

In addition to the demographic information, data collected included: body mass index (BMI), comorbidities (type 2 diabetes {T2DM}, obesity), and lab values (Alanine Aminotransferase {ALT}, Aspartate Aminotransferase {AST}, total bilirubin, albumen, platelet count, and Hemoglobin A1C {HBA1C}) and VCTE values (liver stiffness measurement {LSM}, CAP) were collected. Our cohort consisted of 1244 subjects with evidence of MASLD. The Median age was 52.5 (50.7–54.2) years, 54.9 (48.6–61.2) were male, and median BMI was 32.7 (31.8–33.6) kg/m2. 64.2% of patients were of White race (58.9–69.2), 17.1% Hispanics (12.9–22.3) and 8.8% Black (5.9–13). 36.2% of patients tended to have T2DM (31.8–40.8) whereas 59.4% were obese (53.8–64.8). The characteristics of the study participants is shown in Table 1.

**Table 1:** Baseline characteristics of study participants

Proportion (95%CI)	Overall
**Age**	52.5(50.7–54.2)
**Sex**	
Female	45.1(38.8–51.4)
Male	54.9(48.6–61.2)
**BMI**	32.7(31.8–33.6)
**Race**	
White	64.2(58.9–69.2)
Black	8.8(5.9–13)
Hispanic	17.1(12.9–22.3)
Other	9.9(7-13.7)
**Comorbidities**	
T2DM	36.2(31.8–40.8)
Obesity	59.4(53.8–64.8)
**Lab values**	
Total bilirubin (mg/dL)	0.5(0.47–0.53)
AST (IU/L)	21.88(21.02–22.74)
ALT (IU/L)	25.05(23.6-26.49)
Albumin (g/dL)	4.02(3.97–4.07)
Platelet count (10^3^ cells/uL)	244.65(236.31-252.99)
HbA1C (%)	6.1(5.99–6.22)
LSM (kPa)	6.45(5.88–7.02)
CAP (dB)	308.97(304.98-312.97)
FAST score	0.15(0.14–0.16)

#### Outcome measures

We calculated AGILE 3 + score based on the equation:

$$\eqalign{& Agile{\mkern 1mu} 3{\mkern 1mu} + {\mkern 1mu} = \cr & {\mkern 1mu} {{{e^{\matrix{{ - {\kern 1pt} 3.92368{\kern 1pt} + {\kern 1pt} 2.29714{\kern 1pt} \times {\kern 1pt} \ln {\kern 1pt} \left( E \right){\kern 1pt} - {\kern 1pt} 0.00902{\kern 1pt} \times {\kern 1pt} PLT{\kern 1pt} - {\kern 1pt} 0.98633{\kern 1pt} \times {\kern 1pt} {{ALT} \over {AST}}} \cr { + {\kern 1pt} 1.08636{\kern 1pt} \times {\kern 1pt} Diabete{\kern 1pt} status{\kern 1pt} - {\kern 1pt} 0.38581{\kern 1pt} \times {\kern 1pt} Gender{\kern 1pt} + {\kern 1pt} 0.03018{\kern 1pt} \times {\kern 1pt} Age} \cr } }}} \over {1{\mkern 1mu} + {\mkern 1mu} {e^{\matrix{{ - {\kern 1pt} 3.92368{\kern 1pt} + {\kern 1pt} 2.29714{\kern 1pt} \times {\kern 1pt} \ln {\kern 1pt} \left( E \right){\kern 1pt} - {\kern 1pt} 0.00902{\kern 1pt} \times {\kern 1pt} PLT{\kern 1pt} - {\kern 1pt} 0.98633{\kern 1pt} \times {\kern 1pt} {{ALT} \over {AST}}} \cr { + {\kern 1pt} 1.08636{\kern 1pt} \times {\kern 1pt} Diabete{\kern 1pt} status{\kern 1pt} - {\kern 1pt} 0.38581{\kern 1pt} \times {\kern 1pt} Gender{\kern 1pt} + {\kern 1pt} 0.03018{\kern 1pt} \times {\kern 1pt} Age} \cr } }}}} \cr}$$

We analyzed the prevalence of AGILE 3 + based on the cutoff criteria < 0.45 as rule out, 0.45–0.68 as indeterminate, and ≥ 0.68 as rule in to identify advanced fibrosis in MASLD patients.

Furthermore, using the same population, we calculated AGILE 4 score based on the equation:

$$\eqalign{& Agile{\mkern 1mu} 4{\mkern 1mu} = \cr & {\mkern 1mu} {{{e^{\matrix{{7.50139{\kern 1pt} - {\kern 1pt} 15.42498{\kern 1pt} \times {\kern 1pt} {1 \over {\sqrt E }}{\kern 1pt} - {\kern 1pt} 0.01378{\kern 1pt} \times {\kern 1pt} PLT{\kern 1pt} - {\kern 1pt} 1.41149{\kern 1pt} } \cr { \times {{ALT} \over {AST}}{\kern 1pt} - {\kern 1pt} 0.53281{\kern 1pt} \times {\kern 1pt} Gender{\kern 1pt} + {\kern 1pt} 0.41741{\kern 1pt} \times {\kern 1pt} Diabete{\kern 1pt} status} \cr } }}} \over {1{\mkern 1mu} + {\mkern 1mu} {e^{\matrix{{7.50139{\kern 1pt} - {\kern 1pt} 15.42498{\kern 1pt} \times {\kern 1pt} {1 \over {\sqrt E }}{\kern 1pt} - {\kern 1pt} 0.01378{\kern 1pt} \times {\kern 1pt} PLT{\kern 1pt} - {\kern 1pt} 1.41149{\kern 1pt} } \cr { \times {\kern 1pt} {{ALT} \over {AST}}{\kern 1pt} - {\kern 1pt} 0.53281{\kern 1pt} \times {\kern 1pt} Gender{\kern 1pt} + {\kern 1pt} 0.41741{\kern 1pt} \times {\kern 1pt} Diabete{\kern 1pt} status} \cr } }}}} \cr}$$

We analyzed the prevalence of AGILE 4 based on the cutoff criteria < 0.25 as rule out, 0.25–0.57 as indeterminate, and ≥ 0.57 as rule in to identify cirrhosis in MASLD patients. Appropriate survey weights were applied for all analyses which were performed using Stata version 17 (StataCorp. 2021. Stata Statistical Software: Release 17. College Station, TX: StataCorp LLC.).

## Results

### The prevalence of Advanced Fibrosis utilizing AGILE 3 + score

Based on AGILE 3+, 80.3% (95% CI: 77.1–83.2) of the MASLD population were at low risk for AF and 11.5% (9.2–14.5) were in the grey zone. The overall prevalence of AF due to MASLD was 8.1% (6.2–10.6) corresponding to 4.5 million Americans. Compared to those without AF, the rule-in population tended to be older (64.4 vs. 49.6 years), have higher BMI (37.9 vs. 32), significantly higher T2DM (86% vs. 24.3%) and obesity (80.1% vs. 56.4%) prevalence. There was no significant gender pattern preference, and patients of White race seemed to be most likely to be affected. Those with AF tended to have slightly higher AST (26.04 vs. 21.13 IU/L) and hemoglobin A1C (6.78 vs. 5.92%) but remarkably lower platelet count (201.69 vs. 252.21 per 10^3 cells/uL). Moreover, those with AF had significantly higher LSM measurement (16.65 vs. 5.25 kPa), CAP (331.38 vs. 304.61 dB) and FAST score (0.35 vs. 0.12) compared to those without. The characteristics of AGILE 3 + stratified patients are shown in Table 2.

**Table 2:** Characteristics of patients according to AGILE 3 + score stratification

	AGILE 3+			
	Rule-Out	Indeterminant	Rule-In	*p* value
**Age**	49.6(47.8–51.4)	63.9(60.4–67.4)	64.4(59.5–69.4)	< 0.001
**Sex**				0.989
Female	45.2(38.8–51.7)	44.6(32.2–57.8)	44.4(30.7–59.1)	
Male	54.8(48.3–61.2)	55.4(42.2–67.8)	55.6(40.9–69.3)	
**BMI**	32(31.2–32.8)	33.9(31.4–36.4)	37.9(35.5–40.3)	< 0.001
**Race**				0.233
White	63.6(57.8–69.1)	69.1(60.2–76.8)	63(51.9–72.8)	
Black	8.8(5.9–12.9)	10.5(6.3–17)	6.6(4.1–10.6)	
Hispanic	17.6(13-23.4)	13.9(9-20.7)	16.4(11.6–22.5)	
Other	9.9(6.7–14.5)	6.5(4.1–10)	14(6.9–26.4)	
**Comorbidities**				
T2DM	24.3(20-29.2)	83.7(71.4–91.3)	86(68.7–94.5)	< 0.001
Obesity	56.4(50.6–62)	65.6(53.1–76.2)	80.1(70.7–87)	0.001
**Lab values**				
Total bilirubin (mg/dL)	0.5(0.46–0.53)	0.49(0.42–0.56)	0.53(0.47–0.6)	0.485
AST (IU/L)	21.13(20.14–22.11)	24.17(21.79–26.54)	26.04(20.94–31.15)	0.012
ALT (IU/L)	24.72(23.13–26.3)	26.78(22.25–31.31)	25.85(21.2-30.51)	0.325
Albumin (g/dL)	4.03(3.97–4.09)	3.96(3.86–4.06)	4.02(3.92–4.11)	0.295
Platelet count (103 cells/uL)	252.21(243.73-260.68)	222.31(206.47-238.15)	201.69(190.78–212.6)	< 0.001
HbA1C (%)	5.92(5.79–6.05)	6.91(6.62–7.19)	6.78(6.49–7.07)	< 0.001
LSM (kPa)	5.25(5.08–5.41)	7.67(6.86–8.47)	16.65(12.11–21.18)	< 0.001
CAP (dB)	304.61(300.22–309)	323.56(312.76-334.35)	331.38(315.9-346.86)	< 0.001
FAST score	0.12(0.11–0.13)	0.23(0.18–0.28)	0.35(0.29–0.41)	< 0.001

### The prevalence of MASLD cirrhosis utilizing AGILE 4 score

Based on AGILE 4 score, 96.5% (95% CI: 94.8–97.6) of the MASLD population were at low risk for MASLD cirrhosis and 2.4% (95% CI: 1.4–4.1) were in the grey zone. The overall prevalence of MASLD cirrhosis was 1.1% (95% CI: 0.5–2.3) corresponding to 610,000 Americans. Compared to those without MASLD-cirrhosis, the rule-in population tended to be older (54.5 vs. 52.3 years), have higher BMI (44.7 vs. 32.4), significantly higher T2DM (85.1% vs. 34.9%) and obesity (95.6% vs. 58.3%) prevalence. Men seemed to be more likely to have cirrhosis (56.3% vs. 43.7%) and the patients of White race seemed to be most likely to be affected. Those with cirrhosis tended to have slightly higher AST (27.89 vs. 21.5 IU/L). Cirrhosis patients also had slightly higher hemoglobin A1C (6.54 vs. 6.09%) but remarkably lower platelet count (175.8 vs. 246.71 per 10^3 cells/uL). Total bilirubin (0.6 vs. 0.5 mg/dL) and albumen values (3.91 vs. 4.02 g/dL) were not significantly different between the two groups. Moreover, those with MASLD cirrhosis had significantly higher LSM measurement (37.22 kPa) and FAST score (0.52 vs. 0.15) compared to those without, and higher CAP (318.73 vs. 307.75 dB). The characteristics of AGILE 4 stratified patients are shown in Table 3.

**Table 3:** Characteristics of patients according to AGILE 4 score stratification

	AGILE 4			
	Rule-Out	Indeterminant	Rule-In	*p* value
**Age**	52.3(50.4–54.1)	59(48.8–69.2)	54.5(44.6–64.4)	0.129
**Sex**				0.156
Female	45.6(39.4–52)	24.2(10.1–47.4)	43.7(15.2–77)	
Male	54.4(48-60.6)	75.8(52.6–89.9)	56.3(23-84.8)	
**BMI**	32.4(31.5–33.3)	39.9(34.8–45)	44.7(36.7–52.8)	< 0.001
**Race**				0.118
White	64.5(59.1–69.6)	61.7(48.7–73.3)	43.5(12.5–80.7)	
Black	8.9(6-13.2)	7.2(2.9–17)	2.6(0.3–20.8)	
Hispanic	17(12.7–22.3)	20.8(13.3–31)	17.5(6.2–40.5)	
Other	9.5(6.7–13.4)	10.3(3.6–26.1)	36.4(7-81.2)	
**Comorbidities**				
T2DM	34.9(30.5–39.6)	62.9(29.5–87.4)	85.1(53-96.7)	0.011
Obesity	58.3(52.5–63.9)	88.7(74.3–95.5)	95.6(65.9–99.6)	< 0.001
**Lab values**				
Total bilirubin (mg/dL)	0.5(0.5–0.5)	0.5(0.5–0.6)	0.6(0.4–0.8)	0.509
AST (IU/L)	21.5(20.72–22.27)	34.36(16.52–52.19)	27.89(20.26–35.52)	0.082
ALT (IU/L)	24.8(23.35–26.26)	34.66(19.72–49.59)	25.62(17.41–33.83)	0.236
Albumin (g/dL)	4.02(3.97–4.08)	3.94(3.74–4.14)	3.91(3.54–4.29)	0.432
Platelet count (103 cells/uL)	246.71(238.11-255.32)	193.35(179.33-207.36)	175.8(142.11-209.49)	< 0.001
HbA1C (%)	6.09(5.96–6.21)	6.59(5.97–7.21)	6.54(5.61–7.47)	0.117
LSM (kPa)	5.75(5.5–6.01)	20.55(14.46–26.63)	37.22(21.63–52.81)	< 0.001
CAP (dB)	307.75(303.8-311.71)	353.34(333.08–373.6)	318.73(303.89-333.56)	0.011
FAST score	0.14(0.12–0.15)	0.53(0.42–0.64)	0.52(0.29–0.74)	< 0.001

## Discussion

The noninvasive blood tests such as APRI, NFS and FIB-4 are highly effective at ruling out advanced fibrosis and cirrhosis in MASLD patients but have a relatively low specificity and PPV [9]. Several studies have shown that elastography measurements (CAP, LSM) significantly improve the diagnostic accuracy for MASLD. For example, a prospective analysis of MASLD patients by Eddowes et al. demonstrated an increased prevalence for identifying liver steatosis and fibrosis using FibroScan, thereby showing increased negative predictive values (AURCO 0.87) [10]. Another study by Siddiqui et al. showed that the diagnostic accuracy of VCTE in detecting adults with MASLD accurately distinguishes advanced vs. earlier stages of fibrosis using liver histology as the standard reference [11]. Elastography is more sensitive and specific than traditional ultrasound in detecting fatty liver in the presence of moderate and high probability for fibrosis, however; combining it with blood biomarkers can further enhance the diagnostic accuracy [12, 13].

AGILE 3 + and AGILE 4 scores are an amalgam of elastography measurements (LSM), lab chemistry values (transaminases, platelets) and clinical parameters (presence of diabetes, gender and age), and are thus an attempt to enhance the overall accuracy of diagnostic testing for advance fibrosis and cirrhosis among MASLD patients, respectively. The incorporation of clinical parameters such as diabetes, gender and age into AGILE scores stems from the blatant evidence of their contribution to pathogenesis [14]. Furthermore, AGILE scores utilize a “dual cut-off algorithm” approach which has been shown to correctly classify advanced fibrosis and cirrhosis in hepatitis B patients, reduce the indeterminate grey zone and the false positive rate, and hence reducing the need for liver biopsy [15].

Similar “combining scores” have also been previously developed. For example, Newsome et al. developed FAST score by combining elastography measurements (LSM and CAP) and lab values (AST, ALT, or AST: ALT ratio) [16]. One of the shortcomings of this scoring system is that unlike AGILE scores, FAST score does not take clinical parameters into account. Furthermore, it primarily focuses on patients with fibrosis stage 2 or higher, whereas F ≥ 3 is more clinically relevant. In our study, we aimed to estimate the prevalence of MASLD-related advanced fibrosis and cirrhosis using the AGILE 3 + AGILE 4 scores; respectively, in a U.S. representative sample. In this study of 1244 patients with evidence of MASLD, the overall prevalence of AF due to MASLD based on AGILE 3 + score was 8.1%, corresponding to 4.5 million Americans, whereas based on the AGILE 4 score, the overall prevalence of MASLD cirrhosis was 1.1%, corresponding to 610,000 Americans.

The AGILE scoring system is a non-proprietary test and can be calculated using routinely collected clinical, elastography and laboratory parameters. It is crucial to balance the accuracy of the diagnostic tools and the feasibility of obtaining the diagnostic information in population-based epidemiological studies like this.

### Limitations

The limitations of our study stem mainly from the nature of the AGILE scoring system itself. For instance, individuals who fall under the grey zone (11.5% using AGILE 3 + and 2.4% using AGILE 4) require further evaluation for the presence of MASLD cirrhosis, and therefore evaluating the cost-effectiveness of using AGILE3 + and AGILE 4 as a screening tool in high-risk populations is needed before implementing in clinical practice. Furthermore, despite using a nationally representative sample (NHANES database), the question whether the selected population truly represents the U.S population after applying the inclusion and exclusion criteria remains difficult to answer. The cut off for steatosis () was used based on published literature to maximize sensitivity [17], and different cut off may be used in the clinical settings.

## Data Availability

Datasets used in this analysis can be found online on NHANES database website (https://wwwn.cdc.gov/nchs/nhanes/) for free public access.

## Abbreviations

MASLD: Non-alcoholic liver disease
ALD: Alcoholic liver disease
VCTE: vibration-controlled transient elastography
NHANES: The National Health and Nutrition Examination Survey
NCHS: National Center for Health Statistics
CDC: Center for Disease Control and Prevention
LSM: Liver stiffness measurement
CAP: Controlled attenuation parameter
ALT: Alanine Aminotransferase
AST: Aspartate Aminotransferase
BMI: Body Mass Index {BMI}
HBA1C: Haemoglobin A1C
